# Gastrointestinal Stromal Tumor and Ki-67 as a Prognostic Indicator

**DOI:** 10.7759/cureus.20868

**Published:** 2022-01-01

**Authors:** Kevin J Kadado, Oaklee L Abernathy, William J Salyers, K. James Kallail

**Affiliations:** 1 Internal Medicine, University of Kansas School of Medicine, Wichita, USA

**Keywords:** c-kit, dog-1, mesenchymal tumors, ki-67 expression, gastrointestinal stromal tumor (gist)

## Abstract

Gastrointestinal stromal tumors (GISTs) albeit rare, are the most common mesenchymal neoplasms of our gastrointestinal (GI) tract. GISTs present with nonspecific symptoms and are found incidentally on endoscopy or imaging. A significant portion of GIST diagnoses expresses KIT/CD117 and DOG-1 tissue markers which are pathognomonic for GIST. More recently, Ki-67 was found to be a significant prognostic marker for determining the risk of recurrence. We present a patient with a mesenchymal mass in the small intestine with pathognomonic features of GIST and expression of Ki-67, an important immunocytochemical marker of proliferation.

The patient was a 71-year-old male with a history of hyperlipidemia and hypertension. He presented to the emergency department complaining of bloody diarrhea for two days, with associated nausea, vomiting, and abdominal cramping. Initial blood pressure on presentation was 77/52 mm Hg. Computed tomography (CT) of the abdomen and pelvis revealed a large solid mass with cystic components. The mass was not visualized with esophagogastroduodenoscopy or colonoscopy, and surgical intervention was warranted. A 14 cm x 11.5 cm x 10 cm tumor was found in the ileum. The tumor was excised with small bowel segmental resection and the specimen was sent for pathological evaluation. Immunohistochemical analysis confirmed the diagnosis of GIST with diffuse CD117/c-Kit protein expression. The tumor was high grade with a high mitotic rate at 30 mitoses/50 high-power fields (HPF) and had spindle cell morphology. Of note, 10% of the tumor cells were positive for Ki-67.

GISTs have a high risk of recurrence and a more favorable prognosis with advancements in management. Prior to imatinib therapy in the early 2000s, GISTs prognosis was very poor, as they are resistant to most conventional chemotherapeutic agents and radiation. While the prognosis is fair, surgical resection and imatinib therapy have improved outcomes and risk of recurrence. Prognosis and risk of recurrence can be determined by assessing the mitotic rate, tumor size, and recently, expression of Ki-67. Ki-67 provides a reliable and reproducible approach to assess the prognosis of GIST.

## Introduction

Gastrointestinal stromal tumors (GISTs), first discovered in the 1980s, did not become significant till the 21st century [1]. GISTs are known to be the most common mesenchymal neoplasm of our gastrointestinal tract (GI) [2]. The symptoms tend to be nonspecific and can include nausea, vomiting, early satiety, bloating, fatigue secondary to anemia, abdominal pain, and gastrointestinal bleeding [1-3]. They are also known to be resistant to most conventional chemotherapy agents and radiation treatments [2].­­ The discovery of the c-Kit (tyrosine kinase receptor) mutation in 1998 pioneered the way towards successful treatment of GISTs with imatinib in 2002 [1,2]. GISTs are considered to be rare as there are only seven reported cases per 1,000,000 people in the US [1]. When diagnosing and managing GISTs there are several important diagnostic and prognostic tissue markers that have been reported, including KIT, DOG-1, and more recently, Ki-67. We present a patient with a mesenchymal mass in the small intestine with pathognomonic features of GIST and expression of Ki-67.

## Case presentation

The patient was a 71-year-old male with a history of hyperlipidemia and hypertension. He presented to the emergency department (ED) complaining of bloody diarrhea for two days, with associated nausea, vomiting, and abdominal cramping. His presenting blood pressure was 77/52 mmHg.

**Figure 1:**
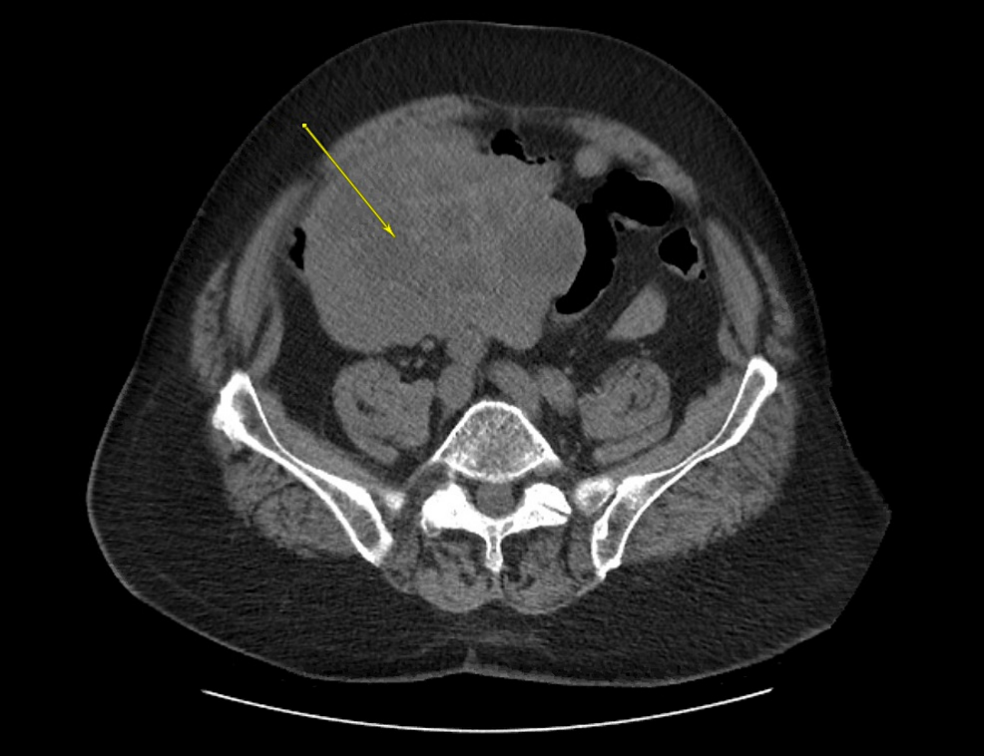
CT abdomen and pelvis The yellow arrow shows a lesion measuring 13.4 cm x 11.2 cm in the lower abdomen

CT of the abdomen and pelvis was significant for a large solid mass (13.4 cm x 11.2 cm), with cystic components in the lower abdomen, located slightly eccentric on the right (Figure 1). Esophagogastroduodenoscopy revealed a mild Schatzki ring, irregular Z-line, and a hiatal hernia with no evidence of ulceration. During the colonoscopy, extensive amounts of hematin were found throughout the entirety of the colon and distal portion of the ileum. During the hospital course, the patient’s hemoglobin was unstable and dropped from 11.4 to 6.6 g/dl within three days. After stabilizing the patient, he was referred to surgery for an exploratory laparotomy during which a 14 cm x 11.5 cm x 10 cm tumor was found in the ileum. The tumor was excised with small bowel segmental resection and the specimen was sent to pathology.

Pathological evaluation of the resected small bowel tumor was consistent with GIST morphology. Immunohistochemical analysis confirmed our diagnosis of GIST with diffuse CD117/c-Kit protein expression. The tumor was high grade with a high mitotic rate at 30 mitoses/50 high-power fields (HPF) and had spindle cell morphology. The tumor was infarcted with a 3.5 cm x 2 cm disruption of the mass consistent with hemorrhage and ischemia. Of note, 10% of tumor cells were positive for Ki-67. The tumor cells were negative for Desmin, S100, Mart-1, AE1:3, p53 wild-type tumor markers. The patient was discharged home and instructed to follow up on an outpatient basis. No further studies or procedures were deemed necessary at that time.

## Discussion

Our patient presented with an abdominal mass with features that were pathognomonic for GIST, in the setting of an acute GI bleed with nausea, vomiting, and abdominal cramping. GISTs are known to arise from the mesenchymal tissue of the GI tract and most studies specifically report that they arise from the interstitial cells of Cajal, also known as the pacemakers cells of the GI tract [1,2,4,5]. About 60% of GISTs originate in the stomach, while about 30%, originate in the small intestine [1,5]. GISTs are known to be sporadic tumors [1,5]. In some instances, they can be associated with syndromes such as NF-1, Carney triad, and familial gastrointestinal stromal tumors [2,3].

Diagnosing a GIST requires a tissue sample for immunohistochemical and morphological analysis [3]. The safest method to obtain a sample is via endoscopic ultrasound-guided fine-needle aspiration (EUS-FNA) [5]. In most instances, GISTs are viewed endoscopically; however, in situations where the mass is inaccessible endoscopically, an open biopsy or surgical resection is warranted [2,3]. If clinical suspicion for GIST is high, a pre-operative biopsy is not required and surgical resection and biopsy are indicated instead [2]. Morphological features and immunohistochemistry alone are not enough to make the diagnosis of GIST and must coincide (i.e. the tumor must be KIT+ and have spindle cell morphology; however, one or the other is not sufficient for diagnosis) [1,3]. The most specific and sensitive marker for the diagnosis of GIST is the overexpression of KIT/CD117 [1]. The presence of KIT/CD117 mutations was found in greater than 90% of GIST diagnoses [3,5]. DOG-1 is another common marker seen with GIST [1,5,6]. DOG-1 was not present in our case but was found in 98% of GISTs [5]. A biopsy can reveal 3 different morphological patterns, spindle cells, epithelioid and mixed [1,3,4,6]. The morphology of GISTs is predominantly spindle cell, as was the case in our patient, and was reported in about 70% of cases [3,6].

The treatment of GISTs is dictated by size, location, and spread [4]. Under most circumstances, surgical resection is the gold standard for GIST management and offers a permanent cure in about 60% of cases [1-6]. It is imperative to perform segmental resection and assess for negative margins [2,3]. Precaution needs to be taken to avoid tumor rupture during resection and avoid abdominal dissemination, as the risk is high [2-5]. While surgical resection is the gold standard for treatment, medical therapy with imatinib, a KIT (tyrosine kinase) inhibitor, is used to treat inoperable, metastatic, or recurrent GISTs [3]. In cases of large GISTs, imatinib has been used with neoadjuvant chemotherapy to help shrink tumor size with a response rate of 82% [5]. In localized non-metastatic GISTs, it is recommended to start imatinib therapy after the operation as data suggests decreased disease recurrence [4,5].

Surgical treatment is only effective in 60% of cases of localized disease [5]. GIST prognosis is overall fair and poor prognostic indicators include size >5cm, high mitotic rate, and distant metastasis [1,2,7]. The risk of recurrence after resection is significant with five years and 15 years recurrence-free survival in patients with surgically resected GIST at 70.5% and 59.9%, respectively [1]. The mitotic rate stands out as a poor prognostic feature that increases the rate of recurrence after surgical resection [1-3]. Tumor size is an independent risk factor for tumor recurrence [1]. Table 1 describes information regarding risk stratification of GISTs according to tumor size and mitotic count [8].

**Table 1:** Risk Stratification of GISTs GIST = Gastrointestinal stromal tumor; HPF = High-power fields

Risk	Size	Mitotic count
Very low risk	<2 cm	<5/50 HPF
Low risk	2–5 cm	<5/50 HPF
Intermediate risk	<5 cm	6–10/50 HPF
	5–10 cm	<5/50 HPF
High risk	>5 cm	>5/50 HPF
	>10 cm	Any mitotic rate
	Any size	>10/50 HPF

The GIST in our patient was significant for 10% Ki-67 expression. Ki-67 is an important immunocytochemical marker of proliferation for several different tumors [7]. Ki-67 was not a statistically significant prognostic factor for overall survivability with GIST [6]. However, Ki-67 showed a strong correlation with the mitotic index, which is a strong prognostic risk factor for GIST [1,6,7]. While Ki-67 is a good prognostic predictor the criteria to analyze Ki-67 are not fully understood for GIST [7]. Expression greater than 8-10% was significant for high risk of recurrence of GIST [6,7]. Additionally, Ki-67 expression greater than 8% can significantly decrease the efficacy of therapy, while tumor size and mitosis count did not significantly reduce efficacy [7].

In terms of follow up, there are not sufficient data suggesting an appropriate time frame for follow up status post diagnosis and resection of GIST [1,3,4]. Some guidelines suggested six months after diagnosis of GIST [3]. The Canadian Committee of GISTs recommended following resected GISTs every three-months status post resection with CT scans [2].

## Conclusions

With the increasing prevalence of GISTs, it is imperative to understand the necessary steps needed to diagnose and manage GISTs reliably and efficiently. Successfully treating GISTs requires a multi-disciplinary approach, and albeit a rare diagnosis, the risk of recurrence, malignant potential, and poor prognosis makes understanding and treating GIST an important tool for clinicians to have. Given the limited but significant Ki-67 data, future studies should focus on Ki-67’s importance in determining prognosis as Ki-67 provides a reliable and reproducible approach to assess prognosis. Future studies should also focus on compiling guidelines or criteria to allow clinicians to use Ki-67 more accurately and consistently as a prognostic indicator.
